# Foraging Behaviors and Comparative Yield Effects of Bumblebee (*Bombus terrestris* Linnaeus) and Chinese Honeybee (*Apis cerana cerana* Fabricius) to Cherry (*Prunus pseudocerasus* ‘Hongdeng’) in Northern China

**DOI:** 10.3390/insects16090900

**Published:** 2025-08-28

**Authors:** Xunbing Huang, Yueyue Wang, Li Zheng

**Affiliations:** 1 College of Resources and Environment, College of Agriculture and Forestry Science, Linyi University, Linyi 276000, China; xunbingh@163.com; 2 Key Laboratory of Natural Enemies Insects, Ministry of Agriculture and Rural Affairs, Jinan 250100, China

**Keywords:** bees, pollination, foraging behavior, fruit tree, pollination effects

## Abstract

Bee pollination is an indispensable part of agricultural production and a crucial factor in maintaining ecosystem balance and biodiversity. Understanding foraging behavior and pollination effects is essential for the management of bee pollination. Over a two-year experiment, we evaluated the foraging behavior and pollination effects of bumblebee *Bombus terrestris* and Chinese honeybee *Apis cerana cerana* on cherries in orchards. Results showed that all bees exhibited enhanced foraging activity as daytime temperatures rose in early spring. However, the daytime foraging activity of bumblebees differs from that of Chinese honeybees. The number of bumblebees leaving the hive exhibited two peak periods, whereas Chinese honeybees showed only one peak period. Bumblebees had longer working hours and greater pollen-carrying capacity than Chinese honeybees. Undoubtedly, cherries pollinated by bees had higher yields, as indicated by a greater fruit setting rate and yield. Thus, as effective pollinators, their pollination significantly boosts production and presents a viable option for widespread use in cherry cultivation. However, the risk of biological invasion by exotic bumblebees cannot be overlooked before extensive use.

## Simple Summary

Bees are the most widely used managed crop pollinators. This study evaluated the foraging behavior and pollination effect of bumblebee *Bombus terrestris* and Chinese honeybee *Apis cerana cerana* on cherries (*Prunus pseudocerasus* ‘Hongdeng’) in northern China. Bee pollination significantly improved cherry production. The daytime foraging activity and pollen-carrying capacity of bumble bees were different from Chinese honeybees. Bee pollination presents a viable option for widespread use in cherry cultivation during early spring in northern China. However, the risk of biological invasion by exotic bumblebees cannot be overlooked before extensive use.

## 1. Introduction

Efficient pollination is a vital component of tree fruit orchard management and crop production. Bee pollination primarily relies on the pollen transfer capabilities of bumblebees, honeybees, and other bees to help angiosperms complete the double fertilization process [1,2]. Through long-term co-evolution, bees have become the primary pollinators of flowering plants, visiting flowers mainly to collect pollen and nectar, providing essential nutrients for individual and group development. Conversely, flowering plants adeptly use bees as a medium to facilitate pollen transfer and complete the pollination process, ensuring population reproduction [3].

The bumblebee (*Bombus terrestris*), an important pollinator, exhibits characteristics such as low temperature tolerance, a large sound and vibration capacity, and a strong ability to collect resources. It can gather 0.01 g of nectar and 1 million pollen grains in a single outing, demonstrating high pollination value in crop production [4,5,6]. *B. terrestris* has been widely applied in developed countries to pollinate crops like greenhouse tomatoes, peppers, eggplants, fruit trees, and more, yielding significant economic benefits. In Europe, *B. terrestris* pollination can even increase the crop yield by more than 17% [7,8]. Additionally, fruit pollinated by bumblebees is known for its delicious and dense taste, uniform size, and high quality, avoiding the malformation caused by hormone dipping, which results in a high commercial fruit rate [9,10]. Bumblebees also play a crucial role in regulating agricultural practices, limiting chemical pesticide use, reducing hormone residues, and ensuring the safety of agricultural products [11,12]. Therefore, bumblebees possess significant pollination value, and studying their behavioral characteristics and effects on crops can provide a theoretical foundation for their further application.

The honeybee *Apis cerana cerana* Fabricius (1973) is an essential pollinating insect in northern China, characterized by its strong cold resistance, keen sense of smell, powerful pollen transfer capabilities, and a wide range of honey source plants. *A. cerana cerana* is significant for maintaining ecological balance in northern China [13,14]. For instance, it has access to a diverse array of nectar source plants, covering nearly all flowering species. It possesses a strong capability to search, collect, and pollinate scattered nectar sources, particularly aiding the pollination of flowering plants in high-cold areas, as well as in early spring and late autumn in northern China [15,16,17]. In agricultural production, *A. cerana cerana* can produce high-value-added products such as honey and serves as a vital pollinator in China’s agricultural ecosystem. Studies showed that *A. cerana cerana* pollination can boost the yield and quality of economic crops like blueberries, strawberries, kiwis, etc. [18,19,20,21]. Thus, *A. cerana cerana* holds significant pollination utilization value for economic crops in China.

However, bee pollination of economic crops has not gained popularity in many regions of northern China. One significant reason bee pollination is rarely utilized in these areas is due to farmers’ limited understanding. With more data on bee behavior and their impact on crops, this situation can change. Comparative field studies examining the pollination effects of bumblebees (*B. terrestris*) and Chinese honeybees (*A. cerana cerana*) to enhance fruiting rates and production yields are essential and urgent. We conducted this research in Yishui County in northern China. Cherry is an important economic crop in this area. The annual cherry production is up to ~58,900 ton, worth USD 196 million (http://nyj.linyi.gov.cn/ (accessed on 16 June 2024)).

This study aims to evaluate the foraging behavior and pollination effects of *B. terrestris* and *A. cerana cerana* on cherry (*Prunus pseudocerasus* ‘Hongdeng’) in orchards. Our objective is to provide data support for the use of bee pollination in cherry cultivation management in northern China.

## 2. Materials and Methods

### 2.1. Research Site

This research was conducted in Yishui County (35.845° N, 118.295° E) in Shandong province of China. The mean annual temperature in the study area is ~13 °C. Air temperatures can fall to −11 °C in December and reach 36 °C in July. The mean annual precipitation is ~850 mm. These climate characteristics provide an ideal natural environment for the development of cherry cultivation.

### 2.2. Bee Pollination in Cherry Orchards

To investigate the pollination behavior and effects of bumblebees and Chinese honeybees on cherries, a two-year orchard experiment involving cherries that received bumblebee pollination, Chinese honeybee pollination, and natural pollination was conducted.

Nine cherry orchards were selected to carry out the pollination experiment in early spring in 2021 and 2023. Each orchard covered an area of approximately 0.13 ha, and the distance between the orchards exceeded 5 km. The same cherry variety ‘Hongdeng’ (*P. pseudocerasus* ‘Hongdeng’) was planted in all nine orchards for seven years, with each orchard containing about 70 cherry plants. Among these, six orchards were separated and covered with hail prevention nets, each featuring mesh shaped like an isosceles triangle, with a long side of 6 mm and a short side of 3 mm. Each of these six orchards was covered by an 1800 m^2^ rectangular hail prevention net. All hail prevention nets were covered on 10th March in each year before cherry flower blooming. No honeybee or other bees occurred due to the lack of nectar sources and low temperature at this time. Hence, we ensured that orchards enriched with specific bee species only obtained pollination services from this specific bee species. These orchards were utilized for studies on bee pollination in the following experiment. The remaining three orchards were open and uncovered, used for investigation into natural pollination. Identical fertilization, irrigation practices, and fungicide treatments were applied across all orchards, while no insecticides were used during the cherry flowering phase. There were no domesticated bees within a 5 km radius for each of nine cherry orchards. We did not conduct these experiments involving cherries that received bumblebee pollination, Chinese honeybee pollination, and natural pollination in 2022 for the COVID-19 pandemic in this year.

Cherry flowers began to bloom in early April of each year. At this time, the bumblebees (*B. terrestris*) were released into the cherry plants in three of the six orchards covered by hail prevention netting. Each bumblebee hive, purchased from Shandong Lubao Technology Development Co., Ltd. (Jinan, China), contained about 100 healthy bumblebees. One bumblebee hive was placed in each orchard until the end of the flowering phase. One hive of Chinese honeybees was positioned in the center of an open, sunny area in each of the other three orchards covered by hail prevention netting. Healthy, pollinating Chinese honeybees were supplied by Wanfengchao Bee Co., Ltd., located in Linyi, Shandong Province, China, and each hive housed approximately 10,000 honeybees. All bee hives were thoroughly checked for health conditions and the presence of any pests or predators. Each hive was free from diseases and microbes. Every 7 days, a continuous examination of the colonies was performed to assess population conditions and check for pests or diseases, such as Chinese scabrood virus (CSBV) and *Ascosphaera apis*, to ensure all colonies are maintaining healthy state. Dead bees were removed from the hives to prevent contamination. The bee pollination of cherry flowers lasted approximately 20 days in each orchard. The remaining three orchards without hail prevention netting relied on natural pollination, with no bees introduced artificially into the area. The native bees that generally occurred in this study area were Chinese honeybee (*A. cerana cerana*). The observed pollinators in natural pollination orchards mainly included wild insects occurring in early spring, such as the wild Chinese honeybees.

### 2.3. Monitoring of Temperature, Relative Humidity, and Bee Pollination Behavior in Cherry Orchards

To monitor the temperature and relative humidity, a HOBO U23 Pro v2 Temperature/Relative Humidity Data Logger (Onset Computer Corporation, Bourne, MA, USA) was installed at height of 1.5 m above the ground in the center of each bee-pollinated orchard. We also monitored bees’ foraging activities from 6:00 to 18:00 h in orchards with bee release on 2 April 2021. We made hourly observations of all installed beehives in each orchard covered with hail prevention nets and recorded the number of bees leaving the beehive, returning to the beehive, and the returning bees carrying pollen within 10 min from 6:00 to 18:00 h. Hence, total of three bumblebee hives and three honeybee hives were observed and recorded, respectively. Ten bees leaving each beehive in each orchard were randomly selected and tracked visually to observe and record the number of visited flowers per minute per individual and the single flower residence time (s) at the same period using the equipment of counter and stopwatch. The daily working time (h) was the difference between the time of the latest bee return and the earliest bee exit.

### 2.4. Investigation of the Effects of Bee Pollination on Cherry

In each of the nine orchards, four trees were randomly selected (~50 m apart) to survey the effects of bee pollination in 2021. Flower counts in each tree were made on six lateral branches selected from the top (two branches), middle (two branches), and bottom (two branches) of the main stem. These selected branches were marked with strings. The number of flowers, fruit, and malformed fruit on each branch of each selected plant was monitored and recorded to calculate the fruit set rate (%) given by the number of fruits on the cherry plant/the number of flowers, and the malformed fruit rate (%) given by the number of malformed fruit/the number of fruits. After full ripening, eighteen cherry fruits from these marked branches (three fruits per branch) in each tree were randomly collected and weighed (g). After the cherry harvest, the cherry fruit yield (kg per 0.13 ha) and sale value in the nine orchards were recorded in 2021 and 2023 to compare cherry fruit production.

A total of 30 cherry blossoms were randomly selected and covered individually with a net bag before they bloomed in each bee pollination orchard in 2023. The net bag was removed on the day when a flower bloomed, and observation continued. After one bee was observed to visit flowers with pollen on the stamen surfaces, the flowers were collected into tubes and marked. Thirty flowers with unopened anthers near the visited flowers were covered with mesh bags and collected into tubes before pollen began to disperse. In each bee pollination orchard, a total of 30 flowers, each visited by respective one bee, and 30 unvisited flowers with pollen were collected and stored in tubes at −20 °C. Then, the pollen count was checked using the same method described by Sun et al. [22]. The pollen carrying rate was calculated for individual bee species by subtracting the number of pollen remaining in pollinated flowers from the number of unpollinated flowers, then dividing by the number of pollen from unpollinated flowers.

### 2.5. Data Analysis

The Student’s *t*-test was used to compare the number of visited flowers per minute, single flower residence time (s), daily working hours (h), and pollen-carrying rate (%) of bees. One-way analysis of variance (ANOVA) and Tukey’s HSD were used to compare the above variables of cherries treated by three pollination methods. All tests were conducted using SAS version 9.0 after verifying the normality.

## 3. Results

### 3.1. Chinese Honeybee and Bumblebee Pollination Activities in Cherry Orchards

**Figure 1:**
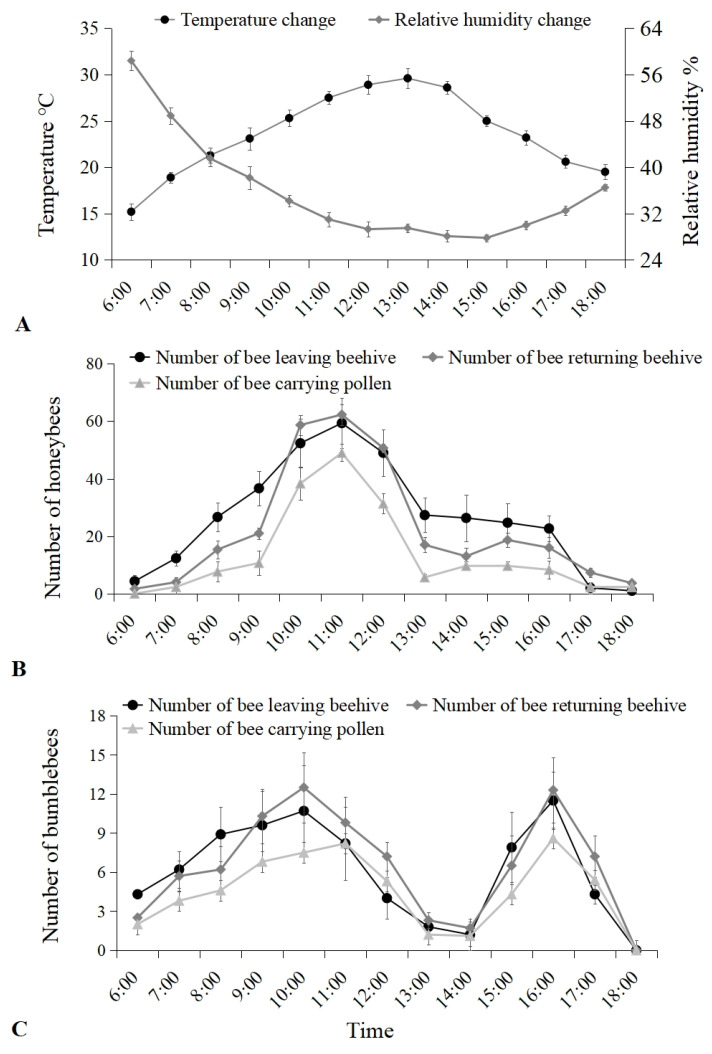
(**A**) The change of temperature (°C) and relative humidity (%), and the number of Chinese honeybees (**B**) and bumblebees (**C**) leaving beehive, returning beehive, and returning bees carrying pollen (individuals per 10 min) in cherry orchards from 6:00 to 18:00 h.

Temperature and relative humidity monitoring (Figure 1A) showed that the overall trend of temperature changes rose from ~16 °C to a maximum of ~30 °C at 13:00 and then decreased to ~20 °C at 18:00. The relative humidity decreased from 55% to a minimum of ~27% at 15:00 and then gradually increased to ~37% by 18:00.

The number of Chinese honeybees leaving the beehive (individuals per 10 min) increased to ~60 individuals from 6:00 to 11:00 h and then gradually decreased to ~1 by 18:00. Similarly, the number of returning bees of carrying pollen (individuals per 10 min) first increased to ~49 individuals from 6:00 to 11:00 h and then gradually decreased to ~2 by 18:00 (Figure 1B).

The number of bumblebees leaving the beehive (individuals per 10 min) exhibited two peak periods. During the first peak period, the number of bees leaving the beehive increased to ~11 individuals from 6:00 to 10:00 h and then gradually decreased to 1 by 14:00. During the second peak period, the number of bees leaving the beehive increased to ~13 individuals from 14:00 to 16:00 h and then gradually decreased to zero by 18:00. Similarly, the number of returning bees of carrying pollen also exhibited two peak periods (Figure 1C).

### 3.2. Pollination Behavior of Bee to Visit Flowers

Monitoring bee behaviors (Table 1) showed that the average number of visited flowers per minute of a single bumblebee and honeybee for cherry was 9.6 and 8.8, respectively. The average single flower residence time of bumblebee and honeybee on cherry was 4.9 s and 5.5 s, respectively. The daily working hours of the bumblebee (10.9 h) were significantly longer (t = 4.595, *p* < 0.05) than the Chinese honeybee (9.8 h) on cherry.

### 3.3. Pollen Carrying Rates of Bumblebees and Chinese Honeybees

**Figure 2:**
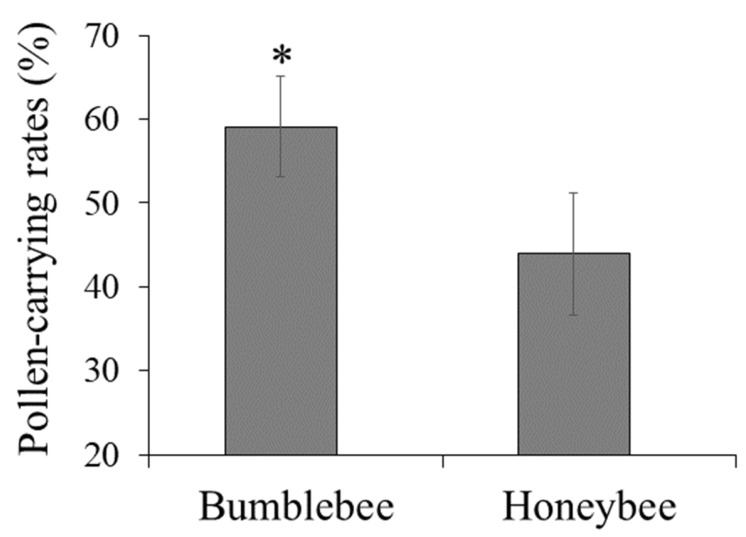
Comparison of pollen-carrying rates of bumblebees and Chinese honeybees. Data are shown as the mean ± SE. * *p* < 0.05 (Student’s *t*-test).

Bumblebees and Chinese honeybees differed in terms of their efficiencies at visiting flowers and carrying pollen. The pollen-carrying rate of bumblebees (59.08%) was higher than Chinese honeybees (43.93%) (t = 4.305, *p* < 0.05) (Figure 2).

### 3.4. Effects of Bee Pollination on Cherry Fruit

**Figure 3:**
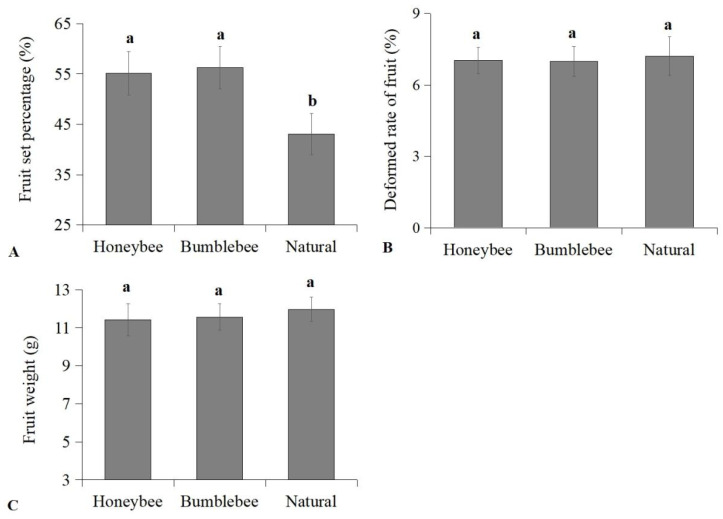
(**A**) Fruit setting rate (% ± SD), (**B**) deformed rate of fruit (% ± SD), and (**C**) fruit weight (g ± SD) of cherry using bumblebee pollination, Chinese honeybee pollination, and natural pollination. Significant differences between three pollination methods are indicated by lowercase letters (ANOVA, Tukey’s HSD analysis, *p* < 0.05).

Results showed that the fruit-setting rate of cherry plants treated with bumblebee pollination (55.15%) and Chinese honeybee pollination (56.27%) was significantly higher compared to natural pollination (43.06%) (Figure 3A) (F = 36.274, *p* < 0.05). The fruit-setting rate increased by 12.09% and 13.21%, respectively. There were no significant differences in fruit weight and the rate of deformed fruit among cherry plants treated with the three pollination methods (Figure 3B,C).

### 3.5. Yield Effects of Bee Pollination on Cherry

**Figure 4:**
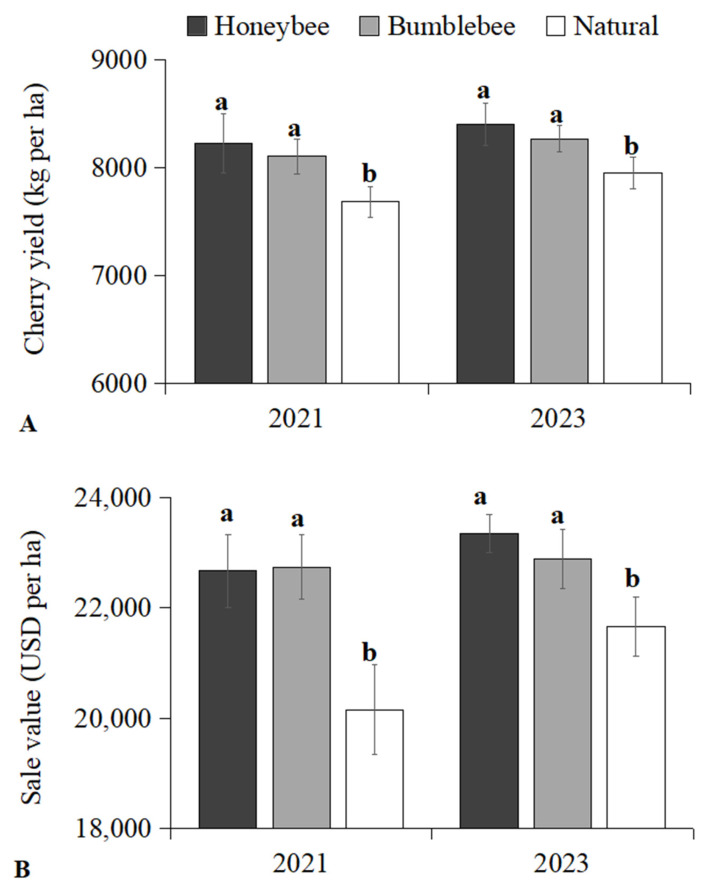
(**A**) Production yield (kg ± SD per ha) and (**B**) sale value of cherry treated with three pollination methods in 2021 and 2023. Significant differences between three pollination methods are indicated by lowercase letters (ANOVA, Tukey’s HSD analysis, *p* < 0.05).

Cherry plants pollinated by Chinese honeybees and bumblebees exhibited significantly higher fruit yield in 2021 (8226 kg per ha, 8101 kg per ha, F = 12.506, *p* = 0.007) and 2023 (8398 kg per ha, 8266 kg per ha, F = 7.167, *p* = 0.026), compared to natural treatment (7679 kg per ha; 7950 kg per ha), respectively (Figure 4A). The fruit yield pollinated by Chinese honeybees increased by 7.12% and 5.64%, respectively, with an average increase of 6.38%. The fruit yield pollinated by bumblebees increased by 5.50% and 3.97%, respectively, with an average increase of 4.73%. Consequently, the recorded sale value of cherry plants pollinated by Chinese honeybees and bumblebees were significantly higher in 2021 (22,671 USD per ha, 22,738 USD per ha, F = 13.581, *p* = 0.006) and 2023 (23,341 USD per ha, 22,883 USD per ha, F = 9.586, *p* = 0.013), compared to natural treatment (20,157 USD per ha; 21,656 USD per ha) (Figure 4B), respectively.

## 4. Discussion

Many factors, including biotic and abiotic elements, can impact bee pollination behaviors. Among these, temperature and humidity changes significantly affect the pollination behavior of bees, with pollination activities peaking under suitable temperature and humidity conditions. In contrast, extreme temperature and humidity reduce efficiency [8,22,23,24]. We observed two peaks in the daytime foraging activity of bumblebees that closely relate to temperature changes from 6:00 to 18:00 h. Bumblebees begin foraging at 6:00 (temperature ~14 °C) in the morning and reach the first activity peak as the temperature increases. However, pollination activity decreases rapidly from 11:00 to 13:00 h when temperatures approach 30 °C, which may hinder bumblebee foraging efforts. After this period, the temperature drops to ~25 °C, resulting in a second peak of foraging activity, which ceases before dark. Therefore, temperatures can directly impact bumblebees’ foraging activities and indirectly affect them by influencing the flowering state of plants. Some studies indicate that temperature can affect anther cracking and pollen release in plant flowers [8,25,26]. With suitable temperatures, some plants secrete more nectar to attract bees. As daytime temperatures rise and relative humidity decreases, the anther cracking of fruit trees increases, releasing a large amount of pollen that provides sufficient honey and pollen sources for bumblebees, encouraging them to forage.

However, the daytime foraging activity of Chinese honeybees is different from that of bumblebees. The number of bumblebees leaving the beehive showed two peak periods, while Chinese honeybees showed one peak period. Similarly, another recent research conducted by Li et al. [27] also indicated that the daily visitation patterns of honeybees (both *A. cerana cerana* and *A. mellifera*) were closely related to the time of day and temperature. Honeybees tended to visit more flowers in the noon and afternoon when the temperature was higher than in the forenoon. The difference in daytime foraging activity between Chinese honeybees and bumblebees may relate to differences in bee sensitivity to temperature changes. Bumblebees are more resistant to low temperatures than Chinese honeybees. In other words, Chinese honeybees have a stronger tolerance for high temperatures. Previous research by Cui and Corlett [28] indicated that the honeybee *A. cerana cerana* was even tolerant of temperatures of 41.5 °C in Yunnan. Detailed studies are needed in the future to better understand these complex relationships between bees, temperature, and relative humidity. In addition, in the present study, we only monitored the number of Chinese honeybees and bumblebees leaving the beehive, returning beehive, and returning bees carrying pollen in cherry orchards. We did not check the number of pollen brought back by an individual bee. To better understand the pollination behavior, the number of pollen brought back by individual bees should be checked to estimate the ‘pollen equivalency’ of these two bee species in further research.

Monitoring of bee behavior showed that bumblebees have longer daily working hours and a stronger pollen-carrying capacity than Chinese honeybees. This may be related to differences in physiological characteristics, behavioral habits, and ecological adaptability between these two bee species. For instance, bumblebees are larger and can carry more pollen. They also have a strong tolerance for harsh environments, such as low light, high humidity, and low temperature, and can release pollen by vibrating flowers [8,12,29,30,31]. Additionally, the foraging behavior of bees is closely related to plant species, particularly the physical or chemical traits of flowers, such as flower color, taste, flowering stage, nutrient level, or volatile organic compounds [24,29,32,33]. Bumblebees may be more sensitive to the physical or chemical traits of cherries and prefer to pollinate them. Further laboratory studies should be conducted to unravel the mechanisms underlying these observations.

Bees possess a remarkable ability to identify active plant pollen, select and collect only high-quality pollen, and visit flowers at the optimal time. These factors enhance the crop fruit-setting rate and yield [8,34]. Undoubtedly, cherries that are pollinated by bumblebees and Chinese honeybees yield higher production levels compared to natural pollination, as evidenced by the increased fruit-setting rate and yield. These findings also align with many recent studies regarding the effects of bumblebees, honeybees, or other wild floral visitors on cherries [35,36,37,38,39] and other crops [12,18,40]. However, in the present study, we only surveyed the number of fruits once to calculate the fruit set rate. In future research, tracking over at least two periods to survey fruits would be better and more accurate. And we acknowledge that we did not explore the composition of pollinator communities and quantify their impact in natural pollination orchards in the present study. Recent research by Eeraerts et al. showed that the pollinators, including honeybees, non-Apis bees, and other insect floral visitors, could jointly enhance the service of pollination to sweet cherry [41,42]. Hence, the composition and impact of pollinator communities, including local wild bees and other insect visitors, should be quantified in natural pollination orchards in future research. As effective natural pollinators, bee activities significantly boost production beyond traditional methods, making them worthy of widespread application in early spring cultivation in northern China. Additionally, we should acknowledge the numerous other benefits of using bee pollination. For instance, bee pollination can reduce labor costs and time investments, thereby enhancing cultivation efficiency in comparison to artificial pollination and hormone treatments. Additionally, it plays a crucial role in regulating agricultural practices, limiting the use of chemical pesticides, minimizing hormone residues, and ensuring the safety of agricultural products. As the area planted with economically important crops increases, bee pollination shows substantial potential in China.

Factors such as climate change, habitat change, and agricultural practices can affect bee populations and their pollination applications [43]. Among these, the irrational use of chemical pesticides by farmers is a significant factor impacting bee pollination applications. Many studies have shown that the unreasonable use of neonicotinoid chemical pesticides, such as imidacloprid and thiamethoxam, has lethal and sublethal effects on bumblebees [44,45,46,47]. Therefore, when utilizing bee pollination in agricultural production, the effects of chemical pesticides should be carefully reconsidered to ensure safety. We can combine bee pollination with biological control technology to achieve a win–win situation in crop pollination and pest control. While using bees for pollination, we can release natural enemies and utilize microbial pesticides.

Recently, the introduction and application of bumblebees, specifically *B. terrestris*, have raised concerns about biological invasion [48]. For example, exotic bumblebees may compete with native bee species for nectar and pollen resources or invade the nests of native bee species, potentially leading to the decline and even extinction of native species. Some exotic bumblebees may carry pathogens, such as parasites and viruses, that can spread to native bee species, resulting in disease outbreaks. These pathogens may be more lethal to native bee species because they lack the corresponding immunity. Additionally, some exotic bumblebees may interbreed with native bumblebee species, causing genetic contamination and impacting the genetic diversity of native bee populations. The hybrid offspring might be more adaptable, further threatening the survival of native bee species. Furthermore, introducing exotic bumblebees may disrupt plant pollination networks, impacting plant reproduction and ecosystem balance. They may favor certain plants, leading to insufficient pollination of others and adversely affecting plant diversity [49,50,51,52]. Therefore, the risk of biological invasion by exotic bumblebees cannot be overlooked. We must adopt scientifically effective measures to harness their pollination benefits while minimizing their adverse effects on the ecosystem. Before introducing exotic bumblebees, a thorough risk assessment should be conducted to evaluate their impact on the native ecosystem. The use of exotic bumblebees should be strictly regulated to prevent their escape. More importantly, we should focus on the domestication and use of native bumblebees for crop pollination to mitigate the risk of biological invasion.

## 5. Conclusions

Our study demonstrates that the daytime pollination activity of bumblebees differs from that of Chinese honeybees. The number of bumblebees leaving the hive exhibited two peak periods, whereas Chinese honeybees showed only one peak period. Bumblebees had longer working hours and greater pollen-carrying capacity than Chinese honeybees. As effective pollinators, bee pollination significantly boosts production and presents a viable option for widespread use in cherry cultivation during early spring in northern China.

## Figures and Tables

**Table 1:** Foraging behavior of bee flower visits on cherry. Significant differences between honeybee and bumblebee are indicated by lowercase letters.

Pollinator	Number of Visited Flowers per Min	Single Flower Residence Time (s)	Daily Working Hours (h)
Honeybee	8.8 ± 0.9	5.5 ± 0.6	9.8 ± 0.4 b
Bumblebee	9.6 ± 1.2	4.9 ± 0.4	10.9 ± 0.5 a

## Data Availability

The data presented in this study are available on request from the corresponding author due to privacy reason.

## References

[1] Vinod M., Naik A.K., Biradar R., Natikar P.K., Balikai R.A. (2016). Bee pollination under organic and conventional farming systems: A review. J. Exp. Zool. India.

[2] Khalifa S.A.M., Elshafiey E.H., Shetaia A.A., El-Wahed A.A.A., Algethami A.F., Musharraf S.G., AlAjmi M.F., Zhao C., Masry S.H.D., Abdel-Daim M.M. (2021). Overview of bee pollination and its economic value for crop production. Insects.

[3] Koetz A.H. (2013). Ecology, behaviour and control of *Apis cerana* with a focus on relevance to the Australian incursion. Insects.

[4] Zhang H., Zhou Z.Y., An J.D. (2019). Pollen release dynamics and daily patterns of pollen-collecting activity of honeybee *Apis mellifera* and bumblebee *Bombus lantschouensis* in solar greenhouse. Insects.

[5] Sun C., Huang J., Wang Y., Zhao X., Su L., Thomas G.W.C., Zhao M., Zhang X., Jungreis I., Kellis M. (2020). Genus-wide characterization of bumblebee genomes provides insights into their evolution and variation in ecological and behavioral traits. Mol. Biol. Evol..

[6] Pashalidou F.G., Lambert H., Peybernes T., Mescher M.C., De Moraes C.M. (2020). Bumble bees damage plant leaves and accelerate flower production when pollen is scarce. Science.

[7] Velthuis H.H.W., Van Doorn A. (2006). A century of advances in bumblebee domestication and the economic and environmental aspects of its commercialization for pollination. Apidologie.

[8] Goulson D. (2010). Bumblebees: Behavior, Ecology, and Conservation.

[9] Huang J., An J., Wu J. (2007). Advantage of bumblebee as pollinator for solanum in greenhouse. Chin. Agric. Sci. Bul..

[10] Zhang H., Huang J., Williams P.H., Vaissière B.E., Zhou Z., Gai Q., Dong J., An J. (2015). Managed bumblebees outperform honeybees in increasing peach fruit set in China: Different limiting processes with different pollinators. PLoS ONE.

[11] Huang X., Li H., Dai X., Wu G., Zhou H., Chen H., Zheng L., Zhai Y. (2021). Research progress of bumblebee behavior and pollination application. Shandong Agric. Sci..

[12] Huang X., Li H., Chen G., Dai X., Zhai Y., Zheng L., Chen H., Zhu P., Ding J. (2022). Pollination behavior of Bombus terristris in winter greenhouse for tomato production in northern China. Shandong Agric. Sci..

[13] Chen B., Luo J. (2023). Research progress of the population genetic differentiation and environmental adaptation mechanisms in *Apis cerana cerana* (Hymenoptera:Apidae). Acta Entomol. Sin..

[14] Wang F., Yang P., Geng J., Xu X. (2007). Conservation and utilization of the Chinese honeybee *Apis cerana* in Bejing. Chin. J. Appl. Entomol..

[15] Wu J. (2012). Honeybee Science.

[16] Wang H., Li L., Chen H., Zhang X., Chen L., Zhao H. (2022). Progress in pollination by *Apis cerana cerana*. Acta Entomol. Sin..

[17] Yu L., Han S. (2003). Effect of habitat and interspecific competition on *Apis cerana cerana* colony distribution. Chin. J. Appl. Ecol..

[18] Zhao D., Su X., Hua Q., Tou L., Chen D. (2019). The comparative studies of pollination behaviors between *Apis cerana* and *Apis mellifera* in blueberry. J. Environ. Entomol..

[19] Xie L., Xue B., Sun Y., Zhao F., Yin J., Geng Y. (2011). The effect of pollination by honeybees on yield of oilseed rape and fatty acid composition of rapeseed. J. Bee.

[20] Han M., Lai K., Zhao Y., Bai F., Li Z., Peng W. (2020). Flower visiting behavior and pollination by honeybees in kiwifruit orchards. Chin. J. Appl. Ecol..

[21] Guo X., Meng C., Feng J. (2023). Diversity of plants foraged by *Apis cerana* based on pollen composition in honey samples in several mountainous areas. Acta Ecol. Sin..

[22] Lyu Z., Zhou T., Sun M., Feng M., Guo W., Nie L., Song Y., Men X., Li L., Yu Y. (2023). Exploratory comparison of flower visiting behavior and pollination ability of mason bees, bumblebees, and honey bees. J. Econ. Entomol..

[23] Whitney H.M., Dyer A., Chittka L., Rands S.A., Glover B.J. (2008). The interaction of temperature and sucrose concentration on foraging preferences in bumblebees. Naturwissenschaften.

[24] Ruedenauer F.A., Spaethe J., Leonhardt S.D. (2015). How to know which food is good for you: Bumblebees use taste to discriminate between different concentrations of food differing in nutrient content. J. Exp. Biol..

[25] Zhao K., Wang Y., Yuan Y., Wei X., Zhang D., Yao D., Zhang J. (2021). Study on the flower-visiting behavior and pollination effect by *Apis cerana cerana* on kiwi fruit. J. Enviro. Entomol..

[26] Wang Y., Tao H., Tian B., Sheng D., Xu C., Zhou H., Huang S., Wang P. (2018). Flowering dynamics, pollen, and pistil contribution to grain yield in response to high temperature during maize flowering. Environ. Exp. Bot..

[27] Li D.F., Tang J., Fan X.D., Chen Y.D., Liu Z., Liang A.T., He Y.T., Yan X.C. (2025). Diurnal temperature and time affect visitation patterns of Honey bees. Sociobiology.

[28] Cui Q., Corlett R.T. (2016). Seasonal and diurnal patterns of activity in honeybees (*Apis* spp.) on the northern edge of the Asian tropics; their implications for the climate-change resilience of pollination. Trop. Conserv. Sci..

[29] Ayasse M., Jarau S. (2014). Chemical ecology of bumble bees. Annu. Rev. Entomol..

[30] Brown M., Brown M.J.F. (2020). Nectar preferences in male bumblebees. Insectes Soc..

[31] Dyer A.G. (2004). Bumblebee search time without ultraviolet light. J. Exp. Biol..

[32] Dyer A.G., Whitney H.M., Arnold S.E.J., Glover B.J., Glover L. (2006). Behavioural ecology: Bees associate warmth with floral colour. Nature.

[33] Witjes S., Eltz T. (2007). Influence of scent deposits on flower choice: Experiments in an artificial flower array with bumblebees. Apidologie.

[34] You C., Chen D., Wu W. (2020). Effects of different pollination methods on yield and quality of greenhouse tomato. J. Changjiang Veg..

[35] Mir M.M., Mir M., Iqbal U., Mushtaq I., Rehman M.U., Iqbal R., Parveze M.U., Khan S.Q., Rather G.H., Banday S.A. (2025). The impact of pollination requirements in sweet cherry: A systematic review. J. Plant Growth Regul..

[36] Osterman J., Mateos-Fierro Z., Siopa C., Castro H., Castro S., Eeraerts M. (2024). The impact of pollination requirements, pollinators, landscape and management practices on pollination in sweet and sour cherry: A systematic review. Agr. Ecosyst. Environ..

[37] Osterman J., Benton F., Hellström S., Luderer-Pflimpfl M., Pöpel-Eisenbrandt A.K., Wild B.S., Theodorou P., Ulbricht C., Paxton R.J. (2023). Mason bees and honey bees synergistically enhance fruit set in sweet cherry orchards. Ecol. Evol..

[38] García C.B., Díaz-Siefer P., Smith-Ramírez C., Montero-Silva F., Martínez-Harms J., Murúa M., Celis-Diez J.L. (2025). Synergistic effect of honeybees and wild floral visitors in promoting sweet cherry fruit set in central Chile. Biol. Res..

[39] McCabe L.M., Boyle N.K., Pitts-Singer T.L. (2024). *Osmia lignaria* (Hymenoptera: Megachilidae) increase pollination of Washington sweet cherry and pear crops. Environ. Entomol..

[40] Li L., Li M., Ni X., Wei M. (2022). Effects of different pollination methods on fruit setting and seed yield of tomato. J. Anhui Agric. Sci..

[41] Eeraerts M., Smagghe G., Meeus I. (2019). Pollinator diversity, floral resources and semi-natural habitat, instead of honey bees and intensive agriculture, enhance pollination service to sweet cherry. Agric. Ecosyst. Environ..

[42] Eeraerts M., Vanderhaegen R., Smagghe G., Meeus I. (2020). Pollination efficiency and foraging behaviour of honey bees and non-Apis bees to sweet cherry. Agr. Forest Entomol..

[43] Goulson D., Lye C.G., Darvill B. (2008). Decline and conservation of bumblebees. Annu. Rev. Entomol..

[44] Smith D.B., Arce A.N., Rodrigues A.R., Bischoff P.H., Gill R.J. (2020). Insecticide exposure during brood or early-adult development reduces brain growth and impairs adult learning in bumblebees. P. Roy Soc. B.

[45] Stanley D.A., Russell A.L., Morrison S.J., Rogers C., Raine N.E. (2016). Investigating the impacts of field-realistic exposure to a neonicotinoid pesticide on bumblebee foraging, homing ability and colony growth. J. Appl. Ecol..

[46] Muth F., Leonard A.S. (2019). A neonicotinoid pesticide impairs foraging, but not learning, in free-flying bumblebees. Sci. Rep..

[47] Bryden J., Gill R.J., Mitton R.A.A., Raine N.E., Jansen V.A.A., Hodgson D. (2013). Chronic sublethal stress causes bee colony failure. Ecol. Lett..

[48] Orr M.C., Ren Z.X., Ge J., Tian L., An J., Huang J., Zhu C.D., Williams P.H. (2022). The rising threat of the invasive bumblebee Bombus terrestris highlights the need for sales restrictions and domestication of unique local biodiversity in Asia. Entomol. Gen..

[49] Rendoll-Carcamo J.A., Contador T.A., Saavedra L., Montalva J. (2017). First record of the invasive bumblebee *Bombus terrestris* (Hymenoptera: Apidae) on Navarino Island, southern Chile (55 S). J. Melittology.

[50] Otterstatter M.C., Thomson J.D. (2008). Does pathogen spillover from commercially reared bumble bees threaten wild pollinators?. PLoS ONE.

[51] Fürst M.A., McMahon D.P., Osborne J.L., Paxton R.J., Brown M.J.F. (2014). Disease associations between honeybees and bumblebees as a threat to wild pollinators. Nature.

[52] Arismendi N., Riveros G., Zapata N., Smagghe G., Gonzalez C., Vargas M. (2021). Occurrence of bee viruses and pathogens associated with emerging infectious diseases in native and non-native bumble bees in southern Chile. Biol. Invasions.

